# Hair cortisol in a naturalistic sample of outpatients with depressive disorders – A secondary analysis of a randomized-controlled trial and a status update on psychoneuroendocrine markers for clinical practice

**DOI:** 10.1016/j.cpnec.2026.100380

**Published:** 2026-09-24

**Authors:** Lena Schindler-Gmelch, Lukas Moses Fuhrmann, Susann Steudte-Schmiedgen, Matthias Berking

**Affiliations:** a Department for Clinical Psychology and Psychotherapy, Friedrich-Alexander-Universität Erlangen-Nürnberg, Nägelsbachstr. 25a, Erlangen, 91052, Germany; b Department of Psychology, TUD Dresden University of Technology, Fetscherstraße 74, Dresden, 01307, Germany; c Department of Psychotherapy and Psychosomatic Medicine, Faculty of Medicine and University Hospital Carl Gustav Carus, TUD Dresden University of Technology, Fetscherstraße 74, Dresden, 01307, Germany

**Keywords:** Hair cortisol, Biomarker, Diagnostic, Intervention-related, Depressive disorders, Affect Regulation Training

## Abstract

**Background:**

Psychoneuroendocrine research has for many years sought diagnostic, prognostic, and intervention-related biomarkers for clinical conditions. Hair cortisol concentrations (HCC) have been considered promising, but findings on their properties as such a biomarker, for instance in depressive disorders, remain mixed.

**Method:**

In a randomized controlled trial (RCT), the Affect Regulation Training (ART), a structured group intervention targeting emotion regulation skills, was compared with a common-factor-control (CFC) and a waitlist control condition (WLC) in an outpatient sample with depressive disorders (major depressive disorder or dysthymia, *N =* 130, *M*_Age_ = 38.65, *SD*_Age_ = 13.28, 73.1% female).

We examined proximal 3 cm hair segments at pre-group-intervention, four-weeks-post-group-intervention/before subsequent individual psychotherapy, and four months after starting subsequent individual psychotherapy. Hair samples were analyzed using chemiluminescence immunoassay (CLIA).

**Results:**

Baseline HCC were neither consistently associated with clinical characteristics nor intervention effects. However, in a subsample rigorously controlled for confounding influences (*n* = 62), HCC had decreased significantly from pre-group-intervention to four months after starting subsequent individual psychotherapy in the ART condition (*p* = .029), but not the CFC and the WLC condition. This was not visible when applying only predefined minimum necessary exclusion criteria (*n* = 82).

**Discussion:**

To our knowledge, this is the first study to demonstrate psychoneuroendocrine changes during a psychotherapeutic intervention in depressive disorders – but only when rigorously controlling for confounding influences, and not accompanied by consistent cross-sectional and predictive results. Building on these and related findings in depressive disorders, we outline the relevance of controlling for confounding influences, as well as key future research avenues to advance psychoneuroendocrinologically informed psychotherapy.

## Highlights

• Hair cortisol decreases in Affect Regulation Training, but not control conditions.
• Hair cortisol not consistently associated with clinical characteristics in depressive disorders.
• Hair cortisol not consistently associated with indicators of treatment success.
• High relevance of rigorously controlling for confounding variables.
• Research avenues discussed for psychoneuroendocrinologically informed psychotherapy.

## 1 Introduction

Psychoneuroendocrinology is a relatively young research field that continues to evolve rapidly. Early growth was driven largely by the laboratories of Dirk Hellhammer and Clemens Kirschbaum, who established salivary cortisol analyses for psychobiological research [e.g., 1], the Trier Social Stress Test as the state-of-the-art laboratory stress protocol [2], and hair analyses for studying long-term endocrine processes (e.g.,[3,4]). These developments were followed by a surge of interest in the 2010s, when psychoneuroendocrine, and particularly cortisol analyses were considered one step away from application in psychotherapeutic practice. Due to the sound underlying theoretical/biological models (e.g., on allostatic load; [5]), the relative ease of sampling (e.g., [4]), and consistent evidence showing (particularly salivary) cortisol reactions to (laboratory) stressors and, thus, as a biomarker for the hypothalamic-pituitary-adrenal (HPA; e.g., [6]) axis, cortisol was soon considered an ideal candidate for diagnostic, prognostic, and intervention purposes to foster biologically informed psychotherapy (e.g., [7]). However, as the available data grew, interpretation became increasingly more complex.

This process is evident in psychoneuroendocrine research on depressive disorders, characterized by depressed mood and lack of interest or pleasure, but often also associated with changes in eating and sleeping and suicidal thoughts or actions (DSM-5; [8]). The clinical applications of psychoneuroendocrine markers here are evident: Accurate assessment of depressive disorders and depressive symptom severity remains a challenge and still largely relies on self-report (i.e., clinical interviews and questionnaires). Further, interventions often rely on a one-size-fits-all, trial-and-error approach, rather than on personalized precision medicine considered essential to advance the field (e.g., [9,10]). Psychoneuroendocrine profiles could provide valuable information for the diagnosis, prognosis, and treatment of depressive disorders and other psychiatric conditions (e.g., [7,11]).

Nevertheless, early studies in depressive disorders utilizing blood, saliva, and urine sampling showed inconsistent results, suggesting positive, negative, or null associations with cortisol levels (reviewed, e.g., [12]). One reason for this heterogeneity may be the temporal resolution of the methods. Blood, saliva, or urine samples provide insights into short-term hormonal secretion over minutes to hours [4,13]. While ideal for laboratory research on acute processes, for long-term changes as in clinical conditions, they are less suited due to their susceptibility to situational influences. Thus, the possibility to analyze endocrine markers from hair samples, providing insights into weeks to months of hormonal secretion, was considered particularly promising for comprehensively understanding long-term psychoneuroendocrine processes in chronic conditions such as depressive disorders (e.g., [3,4]).

However, findings remain mixed: Two studies indicate elevated [14,15], three decreased hair cortisol concentrations (HCC; [[16], [17], [18]]), and eight no differences between individuals with depressive disorders and healthy controls [[19], [20], [21], [22], [23], [24],[24], [25], [26]]. Accordingly, a recent meta-analysis identified no significant group difference and considerable heterogeneity (with only seven of 16 available studies suitable for inclusion, SMD = −0.02, 95% CI: −0.36 - 0.32, I^2^ = 71%, *p* = .002; [27]). Some studies suggest a role of the recurrence of depressive episodes (elevated HCC in the first compared to recurrent episodes and healthy controls; [28,29]), comorbidities (elevated HCC in individuals with comorbid depressive/anxiety disorder; [21], vs. no difference in HCC between individuals with comorbid generalized anxiety disorder/major depressive disorder and healthy controls; [18]), or childhood trauma (higher HCC in individuals with depression in the presence of than in the absence of childhood trauma; [28]; vs. lower HCC in groups with and without depression in the presence of childhood maltreatment; [30]).

Similarly inconsistent results emerge for associations of HCC with depressive symptom severity. Here, two studies reported positive [15,21], two negative [17,24], and six no associations [14,18,20,26,29,30].

For psychotherapeutic interventions, the only available study for depressive disorders, which examined an internet-based intervention, found no significant changes in HCC over the intervention, and no predictive effect of baseline HCC for treatment response [31]. Data on comorbid anxiety and depressive disorders, however, indicated lower pre-treatment HCC in psychotherapy non-responders with respect to depressive symptom severity [32]. Of particular interest would be to examine HCC in the context of standardized, structured, and biologically informed psychotherapeutic interventions – such as the Affect Regulation Training (ART; [33,34]). This group intervention with well-demonstrated clinical efficacy (summarized, e.g., in Ref. [35]) aims to enhance emotion and stress regulation skills and is suitable for individuals with emotion regulation deficits irrespective of diagnosis. Combining techniques from cognitive-behavioral therapy (e.g., Ref. [36]), Dialectical Behavior Therapy [37], mindfulness [38], and similar approaches, it aims to foster affect regulation by addressing the emotional, cognitive, behavioral, and physiological processes involved. For this, it places a particular emphasis on biological mechanisms (i.e., by detailing the hormonal processes of stress and affect and practicing muscle relaxation and breathing techniques).

In a multicenter, prospective randomized controlled trial (RCT, *N* = 218), the efficacy of the ART was examined in an outpatient sample with current depressive disorder (i.e., major depressive disorder or dysthymia; for the study protocol, see Ref. [39]; for the primary outcome data, see Ref. [40]). For this, the ART was contrasted with a common-factor condition (CFC) developed to control for common psychotherapeutic factors not specific to the ART (such as therapeutic alliance, activation of resources, or problem solving, for more detail, see Ref. [39,40]), and a waitlist control condition (WLC). Previously, a marked decrease in depressive symptom severity in the ART compared to the WLC condition could be shown (*d* = 0.56 between pre-intervention and four-week-post-intervention, with the CFC condition in between; [40]). As a secondary point of interest, for a subgroup of *N =* 130 individuals, proximal 3 cm hair segments were collected at three timepoints. The aim of the current study was to examine HCC as a biomarker in this sample over the course of the ART. We tested its properties on a cross-sectional (associations of baseline HCC with baseline clinical characteristics, e.g., depressive symptom severity, number of previous depressive episodes, age at first episode, number and type of comorbidities, emotion regulation skills), predictive (associations of baseline HCC with post-intervention outcomes), and longitudinal level (changes in HCC over the intervention). Due to the lack of pre-registered hypotheses pertaining to HCC analyses and the heterogeneity of findings in the literature, we refrained from stating specific hypotheses here. However, as it is plausible that this heterogeneity is attributable to differences in procedures of data collection and analysis (e.g., controlling for confounding factors, exclusion of cases, for reviews, see, e.g., Ref. [3,41]), we compared two predefined data preprocessing approaches: One applying rigorous exclusion criteria and another applying the minimum exclusion criteria we considered necessary for valid HCC analyses.

## 2 Methods

### 2.1 Participants and procedures

Data was collected between November 2010 and April 2014. Participants were recruited amongst individuals who had applied for outpatient psychotherapeutic care at one of three study centers (Mainz, Marburg, and Kassel, Germany). In an initial telephone screening, inclusion (i.e., age 18 or above, sufficient German language skills) and exclusion criteria (i.e., high risk of suicide, indication of substantial secondary gain such as pending early retirement application, concurrent other psychotherapeutic treatment, acute comorbid psychotic disorder, substance use disorder, bipolar disorder, organic brain disorders or other severe medical conditions, and severe cognitive impairment) were checked. Eligible and consenting individuals were invited to a diagnostic interview (T0) using the Structured Clinical Interview for DSM-IV Axis I and II Disorders (SCID; [42]). Following this, eligible patients with a primary diagnosis of a depressive disorder (i.e., major depressive disorder or dysthymia, *N* = 130, *M*_Age_ = 38.65, *SD*_Age_ = 13.28, 73.1% female) were randomly allocated to the three study conditions stratified by gender (male vs. female), the presence of comorbid diagnoses, previous depressive episodes, and the intake of antidepressant medication (yes vs. no, respectively). This resulted in a sample of *n* = 43 in the ART, *n* = 43 in the CFC (see Ref. [39,40] for a more detailed description), and *n* = 44 in the WLC condition. For further participant characteristics, please see Table 1.

**Table 1:** Demographic, clinical, and hair-related characteristics of the three study conditions following rigorous exclusion criteria for HCC analyses (*n* = 93).

	Intervention condition			Test statistic	*p*-value	Effect size
	ART *n* = 29	CFC *n* = 34	WLC *n* = 30			
**Age (*M*, *SD*)**	38.38 (13.26)	33.15 (12.42)	40.43 (13.14)	*F*(2, 90) = 2.88	0.061	η^2^_p_ = .060
**Female gender (*n*, *%*)**	22 (75.9)	22 (64.7)	21 (70.0)	χ^2^(2) = 0.93	0.629	*V* = 0.100
**Migration background (*n*, *%*)**^a^	1 (3.4)	3 (8.8)	1 (3.6)	χ^2^(2) = 1.16	0.560	*V* = 0.113
**Marital status**				χ^2^(8) = 7.96	0.473	*V* = 0.179
Single (*n*, %)	6 (20.7)	12 (35.3)	5 (16.7)			
In a relationship (*n*, %)	12 (41.4)	13 (38.2)	7 (23.3)			
Married, living together (*n*, %)	6 (20.7)	6 (17.6)	14 (46.7)			
Married, living separately (*n*, %)	2 (6.9)	0 (0)	3 (10.0)			
Divorced (*n*, %)	3 (10.3)	3 (8.8)	1 (3.3)			
**Body Mass Index (BMI; *M*, *SD*)**^b^	25.63 (6.05)	27.62 (4.77)	26.12 (6.15)	*F*(2, 65) = 0.77	0.469	η^2^_p_ = .023
**BMI Categories**^b^				χ^2^(6) = 10.76	0.096	*V* = 0.281
Normal BMI (18.5 – 24.9; *n*, %)	13 (61.9)	8 (33.3)	14 (60.9)			
Overweight (25 – 29.9; *n*, %)	3 (14.3)	10 (41.7)	3 (13.0)			
Obesity Class I (30 – 34.9; *n*, %)	3 (14.3)	5 (20.8)	2 (8.7)			
Obesity Class II (35 – 39.9; *n*, %)	2 (9.5)	1 (4.2)	4 (17.4)			
**Smoking (*n*, *%*)**^c^	4 (19.0)	4 (16.0)	6 (26.1)	χ^2^(2) = 0.78	0.676	*V* = 0.106
**Alcoholic drinks/week (*M*, *SD*)**^d^	2.30 (3.44)	2.46 (2.49)	3.21 (3.18)	*F*(2, 67) = 0.57	0.571	η^2^_p_ = .017
“heavy drinkers” (n, %)^d,e^	2 (5.9)	4 (11.8)	4 (12.1)	χ^2^(2) = 0.93	0.628	*V* = 0.096
**Hair washing frequency: Washes per week (*M*, *SD*)**^f^	4.44 (1.86)	5.15 (1.76)	4.75 (1.88)	*F*(2, 84) = 0.57	0.318	η^2^_p_ = .027
**Physical comorbidities (*n*, %)**^c^	10 (47.6)	9 (36.0)	7 (30.4)	χ^2^(2) = 1.43	0.590	*V* = 0.144
Hypertension (*n*, %)^c^	2 (9.5)	6 (24.0)	1 (4.3)			
Disease of the respiratory system (e.g., asthma, allergy; *n*, %)^c^	2 (9.5)	2 (8.0)	1 (4.3)			
Diabetes (*n*, %)^c^	2 (9.5)	2 (8.0)	0 (0)			
Thyroid Disease (*n*, %)^c^	2 (9.5)	1 (4.0)	2 (8.7)			
Other (*n*, %)^c,g^	4 (19.0)	0 (0)	3 (13.0)			
**Medication (*n*, %)**^h^	9 (45.0)	10 (50.0)	8 (42.1)	χ^2^(2) = 0.25	0.882	*V* = 0.065
Psychiatric medication (*n*, %)^h^	7 (35.0)	8 (40.0)	8 (42.1)	χ^2^(2) = 0.22	0.896	*V* = 0.061
**Primary diagnosis according to SCID (*n*, %)**				χ^2^(12) = 6.47	0.890	*V* = 0.162
F32.0: Mild depressive episode (*n*, %)	4 (13.8)	2 (5.9)	2 (6.7)			
F32.1: Moderate depressive episode (*n*, %)	5 (17.2)	7 (20.6)	3 (10.0)			
F33.0: Recurrent depressive disorder, current episode mild (*n*, %)	7 (24.1)	8 (23.5)	12 (40.0)			
F33.1: Recurrent depressive disorder, current episode moderate (*n*, %)	10 (34.5)	13 (38.2)	11 (37.6)			
F33.2: Recurrent depressive disorder, current episode severe without psychotic symptoms (*n*, %)	2 (6.9)	2 (5.9)	0 (0)			
F33.9: Recurrent depressive disorder, unspecified (*n*, %)	0 (0)	1 (2.9)	1 (3.3)			
F34.1: Dysthymia (*n*, %)	1 (3.4)	1 (2.9)	1 (3.3)			
**Current comorbid diagnoses according to SCID (*n*, %)**	15 (51.7)	20 (58.8)	19 (63.3)	χ^2^(2) = 0.83	0.661	*V* = 0.094
F3: Mood [affective] disorders (*n*, %)	3 (10.3)	2 (5.9)	4 (13.3)			
F4: Neurotic, stress-related and somatoform disorders (*n*, %)	13 (44.8)	16 (47.1)	14 (46.7)			
F5: Behavioural syndromes associated with physiological disturbances and physical factors (*n*, %)	2 (6.9)	5 (14.7)	6 (20.0)			
Other (*n*, %)^i^	0 (0)	0 (0)	1 (3.3)			
**Number of current comorbid diagnoses according to SCID (*M, SD)***	0.66 (0.72)	0.79 (0.85)	0.93 (0.94)	*F*(2, 90) = 0.80	0.451	η^2^_p_ = .018
**Number of previous depressive episodes (*M*, *SD)***^i^	1.52 (1.41)	2.00 (1.81)	2.23 (1.66)	*F*(2, 74) = 1.08	0.346	η^2^_p_ = .575
**Age at first episode (*M*, *SD)***^j^	26.48 (10.03)	25.25 (10.90)	28.48 (12.71)	*F*(2, 75) = 0.55	0.577	η^2^_p_ = .852
**T1 HRSD Score (*M, SD)*^*k*^**	20.92 (8.42)	19.40 (7.02)	19.04 (8.26)	*F*(2, 80) = 0.43	0.653	η^2^_p_ = .011
**T1 BDI-II Score (*M, S)*^*l*^**	30.16 (11.03)	26.75 (11.84)	22.60 (11.51)	*F*(2, 70) = 2.41	0.097	η^2^_p_ = .064
**T1 ERSQ Score (*M, SD)*^*m*^**	45.50 (21.73)	46.19 (18.05)	45.59 (18.13)	*F*(2, 66) = 0.01	0.991	η^2^_p_ < .001
**Season of T1 hair sampling (*n*, %)**				χ^2^(6) = 7.42	0.284	*V* = 0.200
Spring (*n*, %)	8 (27.6)	11 (32.4)	15 (50.0)			
Summer (*n*, %)	8 (27.6)	7 (20.6)	7 (23.3)			
Autumn (*n*, %)	11 (37.9)	13 (38.2)	4 (13.3)			
Winter (*n*, %)	2 (6.9)	3 (8.8)	4 (13.3)			
**Storage time of T1 hair samples in days *(M, SD)***	698.38 (233.82)	733.35 (220.41)	734.17 (229.92)	*F*(2, 90) = 0.24	0.787	η^2^_p_ = .005
**Storage time of T6 hair samples in days *(M, SD)***^n^	607.44 (227.78)	622.16 (216.03)	575.00 (208.35)	*F*(2, 62) = 0.28	0.753	η^2^_p_ = .009
**Storage time of T11 hair samples in days *(M, SD)***^*o*^	506.83 (226.64)	541.94 (210.87)	437.87 (215.71)	*F*(2, 40) = 0.91	0.410	η^2^_p_ = .044
**Batch: % of T1 hair samples analyzed in first batch (*n*, %)**	12 (41.4)	10 (29.4)	9 (30.0)	χ^2^(2) = 1.23	0.546	*V* = 0.115

ART = Affect Regulation Training condition, CFC = common-factor-control condition, WLC = waitlist control condition, BMI = body mass index, SCID = Structured Clinical Interview for DSM-IV Axis I and II Disorders, HRSD = Hamilton Rating Scale for Depression, range = 0 - 75, BDI-II = Beck Depression Inventory II, range = 0 - 63, ERSQ = Emotion Regulation Skills Questionnaire, range = 0 - 108; T1 = before the ten-week group intervention, T6 = ∼four weeks post-intervention (T1 + ∼12 weeks), T11 = ∼four months after the start of individual psychotherapy (T1 + ∼16-19 weeks); ^a^*n* = 29, *n* = 34, *n* = 28, respectively; ^b^*n* = 21, *n* = 24, *n* = 23, respectively; ^c^*n* = 21, *n* = 25, *n* = 23, respectively; ^d^*n* = 22, *n* = 26, *n* = 22, respectively; ^e^ “heavy drinkers” = >14 alcoholic drinks/week for men, >7 alcoholic drinks/week for women according to the National Institute on Alcohol Abuse and Alcoholism [NIAAA]; ^f^*n* = 28, *n* = 33, *n* = 26, respectively; ^g^ Other = musculoskeletal/rheumatological (*n* = 2), gastrointestinal (*n* = 2), dermatological/immunological (*n* = 1), gynecological/endocrinological (*n* = 1), vascular/hematological (*n* = 1) conditions; ^h^*n* = 20, *n* = 20, *n* = 19, respectively; ^i^ Other = F9: Behavioural and emotional disorders with onset usually occurring in childhood and adolescence (*n* = 1); ^j^*n* = 23, *n* = 32, *n* = 22, respectively; ^k^*n* = 26, *n* = 30, *n* = 27, respectively; ^l^*n* = 25, *n* = 28, *n* = 20, respectively; ^m^*n* = 23, *n* = 28, *n* = 18, respectively; ^n^*n* = 18, *n* = 25, *n* = 22, respectively; ^o^*n* = 12, *n* = 16, *n* = 15, respectively.

Following the ART guidelines for an intensive course, 180-min sessions for groups of four to eight participants were conducted once per week for six weeks. After that, participants were invited to independently practice their skills for four weeks, with a 90-min booster session in the eighth week, before they started individual psychotherapy. Hair samples were collected at three timepoints (T1 = before the group intervention, T6 = ∼four weeks post-group-intervention/immediately before the start of individual psychotherapy, T1 + ∼12 weeks, T11 = ∼four months after the start of individual psychotherapy, T1 + ∼16-19 weeks). Participants received €50 for study participation and another €20 for providing hair samples. For a full flowchart on screening, recruitment, and sampling time points and more information on study procedure and further measures, please see the published study protocol [39]. The study was approved by the ethics committee of the German Psychological Society and the University of Marburg, and the primary analyses were pre-registered with ClinicalTrials.gov, number NCT01330485 (https://clinicaltrials.gov/study/NCT01330485).

### 2.2 Measures

Sociodemographic (e.g., age, gender), health-, and hair-related variables (i.e., body mass index [BMI], smoking, alcohol intake, physical comorbidities, medication, hair washing frequency) were assessed via self-developed checklists (please see Table 1 for all assessed information).

Depressive symptom severity was assessed via the 24-item version of the Hamilton Rating Scale for Depression (HRSD; [43]). This clinician-administered, semi-structured interview allows the assessment of depressive symptoms such as depressed mood, feelings of guilt, and sleep disturbances over the past week on three-point or five-point Likert scales, resulting in a total range of 0–75). As a second measure of depressive symptomatology, the Beck Depression Inventory II (BDI-II; [44]) was implemented. Its 21 self-report items (four-point Likert scale, total range: 0-63) provide an overview of the severity of somatic, behavioral, emotional, and cognitive symptoms of depressive disorders over the past two weeks.

Successful application of adaptive emotion regulation skills as the key focus of the ART was assessed using the 27-item Emotion Regulation Skills Questionnaire (ERSQ; [45], five-point Likert scale, total range: 0-108). The ERSQ is based on the emotion regulation model of the ART and assesses the application of the nine competencies Awareness, Sensations, Clarity, Understanding, Acceptance, Tolerance, Readiness to Confront, Self-Support, and Modification over the past week.

Further self-report data on, e.g., emotion regulation and well-being, was collected (cf. study protocol; [39]). However, to avoid inflation of Type I error, we decided to focus on the main study outcomes (i.e., depressive symptom severity) and emotion regulation as the key focus of the ART here.

### 2.3 Hair sample collection and preparation

Hair samples were cut in a standardized fashion as closely to the scalp as possible at the posterior vertex region [4] where hair growth is considered to be most uniform [46]. Two to three hair samples, each with an approximate overall diameter of 3 mm were sampled, wrapped in aluminum foil, stored in a dry, dark environment and sent to the biopsychological laboratory of the TUD Dresden University of Technology. Analysis was done in two batches in June/July 2014 (for mean storage times per assessment timepoint, please see Table 1). For the evaluation of HCC, the 3 cm hair segment closest to the scalp was analyzed, representing a three-month accumulation of hormonal secretion deduced from an estimated monthly hair growth rate of 1 cm [47]. For hair washing and steroid extraction, a slightly modified version of the protocol of [48] was employed. In brief, each hair segment was washed twice with isopropanol in 20 mL glass vials, with the vials gently shaken by hand in an overhead motion. After that, the hair samples were allowed to dry for at least 12 h. The segments were cut, and 7.5 mg of hair (as roughly needed for reliable analyses) was carefully weighed and 1.8 mL of pure methanol added. The vials were covered for 18h for steroid extraction. Then, 1.6 mL of the clear supernatant was transferred into a new 2 mL Eppendorf tube. The alcohol was then evaporated at 60 °C under a constant stream of nitrogen until the samples were completely dry (duration: approximately 20 min). Finally, 0.175 mL of water was added and the tube vortexed for 15 s. Twenty μL were removed and used for cortisol determination with a commercially available immunoassay with chemiluminescence detection (CLIA, IBL, Hamburg, Germany). Sensitivity, specificity, and reliability of this method have been shown, with inter- and intra-assay coefficients of variation below 8%. A subset of hair samples (3.55%) was analyzed in duplicate to assess analytical precision. No sample was outside the detection limit. For a small number of samples (4.8% for T1, 4.2% for T6, and 5.1% for T11), the available hair did not reach the intended mass (i.e., due to the sampled strands being too thin). However, as all samples had a minimum weight greater than 5 mg, we decided to still include them in all further analyses.

### 2.4 Statistical analyses and data exclusion

Statistical analyses were conducted using SPSS for Windows, version 31 [49] and R [50]. Data and analysis code can be found in OSF under https://osf.io/4pkhv/overview?view_only=b7f8b79226a24f039d8522714a4aa479. As HCC data was not normally distributed and log-transformation also did not achieve normality, original data and statistical analyses robust to violations of the normality assumption were used. In order to maximize exploratory power of the HCC analyses, no post-hoc Type I error correction methods were applied.

Due to the characteristics of the study (secondary analysis of an RCT, naturalistic study sample), and the lack of established guidelines on preprocessing of HCC data, exclusion of participants proved to be a complex process. In a first step, we applied predefined strict criteria, matching the most rigorous approaches in clinical HCC research (for an overview, see, e.g., Ref. [3,4,13,41]). We excluded participants with a) an intake of glucocorticoid medication (*n* = 3), b) biologically implausible HCC values following visual inspection (in the case of this study, samples > 500 pg/mg). Further, for individuals with c) outlying values of > 3 SD above the mean of the respective condition and time point (*n* = 2 for T1, *n* = 3 for T6, and *n* = 4 for T11), we utilized fixed-level imputation, replacing them with M +3 SD for the respective condition and timepoint. No outlying values of < 3 SD below the mean of the respective condition and time point were observed. Additionally, we excluded d) a hair length of < 3 cm (*n* = 4 for T1, *n* = 2 for T6, *n* = 2 for T11), e) a BMI ≤ 18 (*n* = 3) or ≥ 40 (*n* = 5), f) individuals smoking > 10 cigarettes per day (*n* = 11), g) consuming > 14 alcoholic drinks/week for men, > 7 alcoholic drinks/week for women following NIAAA's definition of heavy drinking ([51]; *n* = 10), and h) reporting an intake of thyroid or other hormonal medication (*n* = 9). Further, we excluded individuals who dropped out during the intervention while still supplying hair samples and questionnaire data (*n* = 4 between T1 and T6, *n* = 2 between T6 and T11, due to, e.g., symptoms too severe for continued group/outpatient therapy). Together with the attrition for the longitudinal study parts (T6: *n* = 103, T11: *n* = 88), this resulted in a sample of *n =* 93 for T1 (cross-sectional analyses), *n =* 65 for T1 – T6 and *n =* 62 for T1 – T6 – T11 (longitudinal analyses).

In a second step, we conducted the same analyses using predefined broader inclusion criteria that we considered the minimum necessary for valid HCC analyses, thereby maximizing statistical power and preserving the naturalistic character of the sample. For this, we only applied the exclusion criteria a – c) from above. This resulted in a sample of *n =* 126 for T1 (cross-sectional analyses), *n =* 99 for T1 – T6, and *n =* 82 for T1 – T6 – T11 (longitudinal analyses).

The three intervention conditions were compared regarding baseline demographic, health- and hair-related, and clinical data using generalized linear models (GLMs) with Bonferroni-corrected post-hoc tests for continuous and χ^2^ tests of independence for dichotomous variables, respectively. Next, to confirm the results of the primary outcome paper [40] also for the HCC sample, changes in depressive symptom severity (HRSD, BDI-II) over the intervention were assessed with robust linear mixed models with the between-subjects factor “condition (ART, CFC, WLC)”, and the within-subjects factor “time (T1, T6, T11)” using the robustlmm [52] and the emmeans [53] package in R. Further, baseline correlational analyses of HCC with potential confounding factors (i.e., BMI, number of cigarettes per week, number of alcoholic drinks per week, hair washing frequency, season of sampling, storage time of hair samples, batch of analysis) were conducted using Spearman (*r*_sp_) correlation analyses and χ^2^ tests of independence, respectively. Identified confounders were added to all further models as sensitivity analyses.

For baseline associations of HCC with clinical characteristics (i.e., number of previous depressive episodes, age at first episode, number of current comorbid disorders, HRSD sum score, BDI-II sum score, ERSQ sum score), Spearman (*r*_sp_) and partial Spearman correlation analyses were conducted, controlling for the identified confounders. To assess whether clinical features of depressive disorders (first episode yes/no, comorbid anxiety disorder yes/no) were predicted by baseline HCC, logistic regression models were fitted, with potential confounders added as covariates in a second step.

For predictive analyses, robust linear regression analyses were conducted using M-estimators via the packages MASS [54], sandwich [55,56], and lmtest [57] in R. Main clinical outcomes (change scores of HRSD, BDI-II, ERSQ, computed as post-pre-value) were added as dependent and baseline HCC and baseline clinical characteristics as independent variables. Newey–West–adjusted standard errors provided heteroskedasticity- and autocorrelation-robust inference. For predictive analyses on remission status, determined as no longer fulfilling criteria for depressive disorders (i.e., a value ≤ 9 on the HRSD or ≤ 11 on the BDI-II), logistic regressions were applied. To account for heterogeneous processes in the active conditions, analyses were conducted across the two active conditions (ART, CFC) and in the ART condition alone.

In order to analyze changes in HCC over the intervention, robust linear mixed-effects models with the between-subjects factor “condition (ART, CFC, WLC)”, and the within-subjects factor “time (T1, T6, T11)” were implemented using, again, robustlmm and emmeans in R.

## 3 Results

Participants in the three study conditions were well-matched regarding socio-demographic, health-, and hair-related variables at baseline, both for rigorous (all *p* ≥ .061, see Table 1) and minimum necessary exclusion criteria (all *p* ≥ .121, see Supplement A).

With respect to depressive symptom severity changes according to the HRSD over T1 – T6 – T11, both for rigorous and minimum necessary exclusion criteria, post-hoc tests of the robust linear mixed models indicated significant reductions from T1 to T6 (pre-group-intervention until four weeks post-group-intervention) only for the ART (*p* = .027 and *p* = .004) and the CFC (*p* = .002 and *p* < .001), not the WLC group (*p* = .966 and *p* = .183). From T6 to T11 (four weeks post-group-intervention until four months after start of individual psychotherapy), reductions were visible for all three conditions (ART: *p* = .009 and *p* < .001; CFC: *p* = .013 and *p* = .008; WLC: *p* < .001 and *p* < .001). For the BDI-II, similar results emerged, with post-hoc tests again indicating significant reductions from T1 to T6 only for the ART (*p* < .001 and *p* < .001) and the CFC (*p* = .004 and *p* < .001), not the WLC condition (*p* = .420 and *p* = .246), and from T6 to T11 only for the CFC (*p* = .029 and *p* = .001), not the ART or the WLC condition (all *p* ≥ .09).

Regarding potential confounding factors, for rigorous exclusion criteria (*n =* 93), baseline HCC were associated with batch of analysis (*r*_sp_ = .24, *p* = .018). For minimum necessary exclusion criteria (*n =* 126), the association between baseline HCC and batch emerged at trend level, while an additional association with baseline BMI (*r*_sp_ = .22, *p* = .027) was visible. No associations emerged for number of cigarettes per day, number of alcoholic drinks per week, hair washing frequency, season of sampling, or storage time (all *p* ≥ .307). Thus, for the rigorous exclusion criteria sample, batch of analysis, and for the minimum necessary exclusion criteria sample, batch of analysis and BMI were added as covariates in all further analyses.

### 3.1 Associations of HCC with baseline clinical characteristics

With the rigorous exclusion criteria, no associations of baseline HCC with any indicator of baseline clinical characteristics (number of previous depressive episodes, age at first episode, number of current comorbid disorders, HRSD sum score, BDI-II sum score, ERSQ sum score) emerged (all *p* ≥ .124). Further, neither recurrence of depressive disorder nor comorbidity of an anxiety disorder was associated with baseline HCC (all *p* ≥ .327). Sensitivity analyses controlling for batch yielded a singular positive association of baseline HCC with baseline HRSD depressive symptom severity (*r*_*sp*_ = .26, *p* = .027). While this association emerged at trend level also for the minimum necessary exclusion criteria sample (*r*_*sp*_ = .18, *p* = .062), here, it vanished when controlling for batch and BMI (*r*_*sp*_ = .19, *p* = .167).

### 3.2 HCC as an intervention-related biomarker

#### 3.2.1 Predictive analyses

For the rigorous exclusion criteria, linear and logistic regression analyses examining the predictive value of baseline HCC levels for changes in depressive symptom severity (HRSD, BDI-II) and emotion regulation (ERSQ) as well as HRSD and BDI-II remission status over the two active conditions (*n* = 63) and the ART condition alone (*n* = 29) revealed no associations (all *p* ≥ .126, see Table 2). Analyses controlling for batch yielded parallel results (all *p* ≥ .106).

**Table 2:** Robust linear regression analyses on the predictive value of baseline HCC on clinical characteristics at T6 and T11, with baseline clinical characteristics as covariate, for the sample following rigorous exclusion criteria.

Dependent	Predictor	B [KI]	SE	Beta	*p*
T1 – T6: HRSD Change Score – ART & CFC (*n* = 53)	Baseline HRSD Score	−0.66 [-0.95; −0.36]	0.15	−0.55	< .001
	Baseline HCC	0.01 [-0.01; 0.04]	0.01	0.12	0.340

T1 – T6: HRSD Change Score – ART (*n* = 21)	Baseline HRSD Score	−0.10 [-0.45; 0.26]	0.18	−0.10	0.591
	Baseline HCC	0.01 [-0.22; 0.24]	0.12	0.01	0.939

T1 – T11: HRSD Change Score – ART & CFC (*n* = 48)	Baseline HRSD Score	−0.50 [-0.75; −0.25]	0.13	−0.49	< 0.001
	Baseline HCC	0 [-0.02; 0.02]	0.01	0.03	0.787

T1 – T11: HRSD Change Score – ART (*n* = 17)	Baseline HRSD Score	−0.73 [-0.98; −0.48]	0.13	−0.74	< 0.001
	Baseline HCC	−0.11 [-0.24; 0.03]	0.07	−0.19	0.126

T1 – T6: BDI-II Change Score – ART & CFC (*n* = 41)	Baseline BDI-II Score	−0.19 [-0.41; 0.02]	0.11	−0.25	0.076
	Baseline HCC	< 0.001 [-0.02; 0.02]	0.01	0.01	0.965

T1 – T6: BDI-II Change Score – ART (*n* = 18)	Baseline BDI-II Score	−0.54 [-1.01; −0.08]	0.24	−0.49	0.023
	Baseline HCC	0.04 [-0.39; 0.47]	0.22	0.04	0.865

T1 – T11: BDI-II Change Score – ART & CFC (*n* = 42)	Baseline BDI-II Score	−0.34 [-0.62; −0.06]	0.14	−0.33	0.017
	Baseline HCC	0 [-0.02; 0.03]	0.01	0.04	0.776

T1 – T11: BDI-II Change Score – ART (*n* = 14)	Baseline BDI-II Score	−0.51 [-1.13; 0.11]	0.32	−0.53	0.108
	Baseline HCC	−0.06 [-0.49; 0.36]	0.22	−0.10	0.767

T1 – T6: ERSQ Change Score – ART & CFC (*n* = 40)	Baseline ERSQ Score	0.43 [0.16; 0.71]	0.14	0.45	0.002
	Baseline HCC	−0.01 [-0.06; 0.04]	0.03	−0.05	0.727

T1 – T6: ERSQ Change Score – ART (*n* = 16)	Baseline ERSQ Score	0.33 [0.01; 0.65]	0.16	0.46	0.045
	Baseline HCC	0.03 [-0.52; 0.58]	0.28	0.02	0.913

T1 – T11: ERSQ Change Score – ART & CFC (*n* = 39)	Baseline ERSQ Score	0.19 [-0.07; 0.45]	0.13	0.21	0.151
	Baseline HCC	−0.01 [-0.05; 0.04]	0.02	−0.04	0.792

T1 – T11: ERSQ Change Score – ART (*n* = 12)	Baseline ERSQ Score	0.37 [0.11; 0.64]	0.13	0.55	0.005
	Baseline HCC	−0.1 [-0.45; 0.25]	0.18	−0.11	0.575

ART = Affect Regulation Training condition, CFC = common-factor-control condition, HRSD = Hamilton Rating Scale for Depression, BDI-II = Beck Depression Inventory II, ERSQ = Emotion Regulation Skills Questionnaire; T1 = before the ten-week group intervention, T6 = ∼four weeks post-intervention (T1 + ∼12 weeks), T11 = ∼four months after the start of individual psychotherapy (T1 + ∼16-19 weeks).

For minimum necessary exclusion criteria (*n* = 82 over both active conditions, *n* = 42 for ART only), parallel results emerged (all *p* ≥ .100), apart from higher baseline HCC, at trend level, indicating a greater likelihood of BDI-II remission at T6 over both active conditions (*B* = 0.17, *SE* = 0.09, χ^2^ = 3.47, *p* = .063, *OR* = 1.18, see Supplements B and C). Analyses controlling for batch and baseline BMI, again, led to parallel results (trend-level association with likelihood of BDI-II remission at T6: *B* = 0.21, *SE* = 0.11, χ^2^ = 3.77, *p* = .052, *OR* = 1.24; all other *p* ≥ .188).

#### 3.2.2 Changes in HCC over the intervention

For the rigorous exclusion criteria (*n* = 62 for T1 – T6 – T11 analyses), robust linear mixed-effects models showed a significant decrease of HCC from T1 to T11 in the ART condition only (Δ = −3.18, *SE* = 1.45, *z* = −2.19, *p* = .029), which was visible as a trend-level effect already between T1 and T6 (Δ = −2.60, *SE* = 1.37, *z* = −1.89, *p* = .058; see Fig. 1a). No HCC changes emerged in the CFC or the WLC condition (all |z| < 1.33, *p* ≥ .183). For the minimum necessary exclusion criteria (*n* = 82 for T1 – T6 – T11 analyses), these effects did not emerge (all |z| < 1.26, *p* ≥ .206; see Fig. 1b). Analyses controlling for batch (rigorous exclusion criteria) and batch and BMI (minimum necessary exclusion criteria) yielded parallel results.

**Fig. 1:**
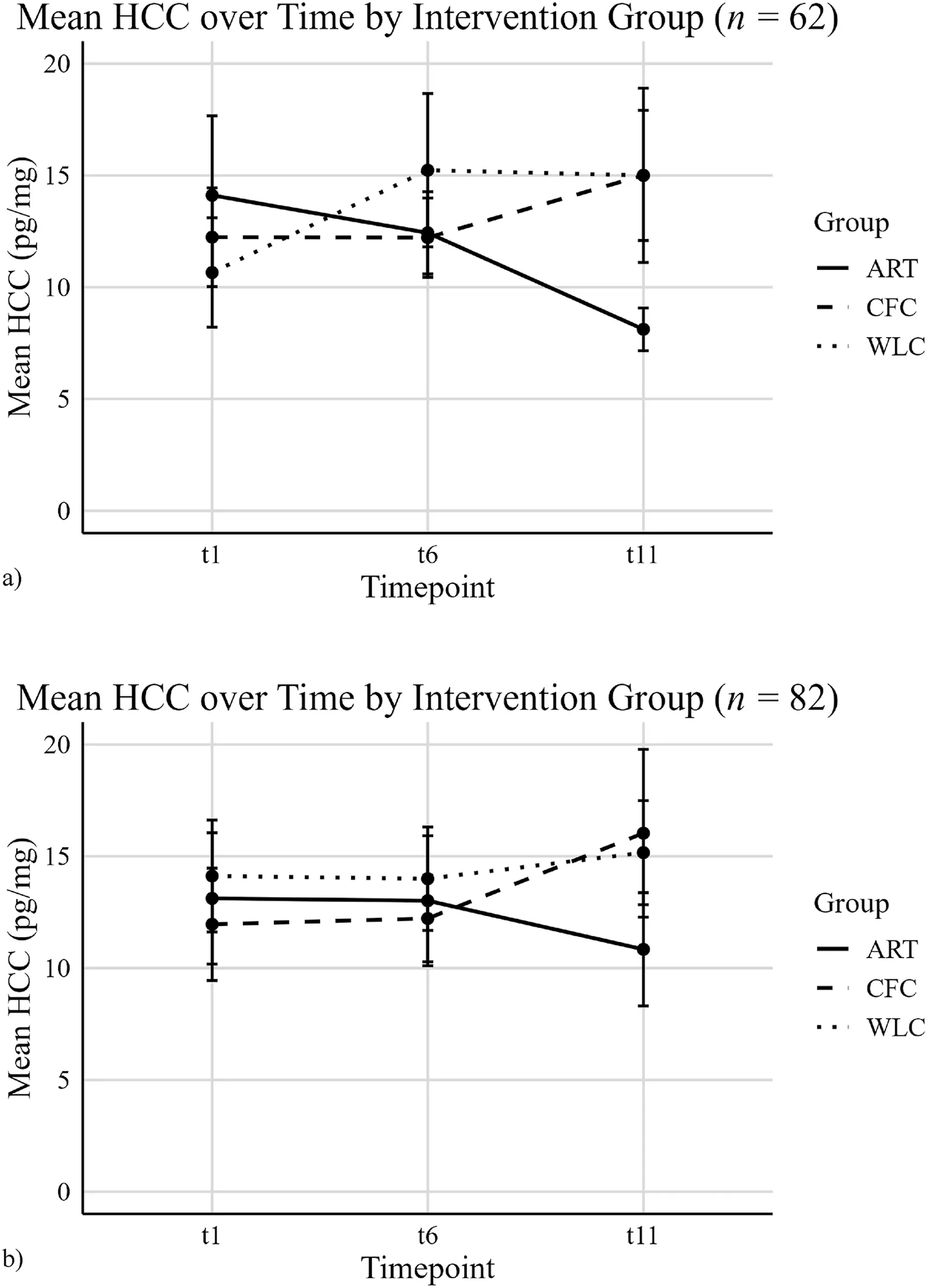
Mean ( ± SEM) hair cortisol concentrations (HCC) over the intervention (T1 = before the ten-week group intervention; T6 = ∼four weeks post-intervention, T1 + ∼12 weeks; T11 = ∼four months after the start of individual psychotherapy, T1 + ∼16-19 weeks); by study condition (ART = Affect Regulation Training condition, CFC = common-factor-control condition, WLC = Waitlist Control Condition); a) following rigorous exclusion criteria (*n* = 62); and b) following minimum necessary exclusion criteria (*n* = 82) for confounding variables.

## 4 Discussion

The aim of our study was to assess HCC as a biomarker for symptom severity and intervention effects in an RCT examining the effectiveness of the ART (vs. CFC and WLC) in depressive disorders. We found no consistent associations of baseline HCC with baseline clinical characteristics (depressive symptom severity and emotion regulation skills, recurrence and comorbidity characteristics), and no consistent predictive associations with clinical improvement. However, notably, when applying rigorous exclusion criteria for potential confounding influences as identified in 15 years of psychoneuroendocrinological research, we saw a significant decrease of HCC in the ART, but not in the CFC or WLC conditions (*n* = 62, *p* = .029). This effect vanished when applying only minimum necessary exclusion criteria (*n* = 82).

The finding of ART-specific HCC changes is remarkable, considering that for the first time, to the best of our knowledge, psychoneuroendocrine changes could be demonstrated for a psychotherapeutic intervention in depressive disorders. This complements the clinical data from the current and the primary outcome sample [40] of a marked decrease in depressive symptom severity in the ART (and, to a lesser extent, the CFC) compared to the WLC condition. It is plausible that the improvements in emotion regulation following ART led to reduced physiological arousal, reflected in attenuated HPA axis activity. Notably, the between-group difference was driven not only by decreases in HCC in the ART group, but also increases in HCC in the WLC and the CFC groups. This pattern points towards a specific superiority of ART components over common factors of psychotherapy such as therapeutic alliance or resource activation. However, the fact that HCC only emerged as an intervention-related biomarker if the most rigorous criteria regarding potential confounders were applied, combined with the observation that HCC were neither consistently associated with baseline clinical characteristics, nor predictive of clinical changes, dampens the enthusiasm. To reconcile these results, two interpretations are plausible:

First, it is possible that our findings indicate existing, meaningful, and specific associations of HCC with treatment success in depressive disorders, and that the heterogeneous results from our analyses, as well as the literature (summarized, e.g., in Ref. [12,27]), are best explained by confounding variables. The available studies greatly differed in methodology, e.g., with respect to participant characteristics (e.g., sex or clinical characteristics) and methodological aspects (e.g., sampling, storage, and analysis techniques). In the current, naturalistic sample, HCC, additionally, were not the primary study outcome. Thus, confounding influences were quite prevalent, which may have masked associations of HCC with clinical characteristics and intervention effects beyond the ones we managed to identify via rigorous exclusion criteria. Arguably, the benefits of a biomarker that requires strict control for confounding influences are greatly limited in clinical practice – in particular for longitudinal assessments over the course of interventions. The following factors, most prominently, might have played a role (and constitute limitations of the current study):

a) Regarding participant characteristics
  - High levels of psychiatric comorbidity, particularly of anxiety disorders (46.2% of participants), may have exerted interacting or moderating effects on depression-related associations. Most prominently, trauma history/childhood trauma was not explicitly assessed. While the SCID captures the presence of posttraumatic stress disorder (PTSD), the resulting subgroup (*n* = 3) was too small for in-depth sensitivity analyses. This is unfortunate, as trauma status/PTSD is known to substantially impact HPA axis functioning (for a systematic review, see Ref. [41]), and also previously was shown to play a role in depressive disorders [28,30]. However, it must be noted that also in trauma/PTSD, the exact mechanisms underlying HPA axis alterations are a long way from being completely understood [41].
  - Considerable prevalence of obesity, heavy nicotine, and alcohol intake. All three factors are considered confounders for HPA axis activity (e.g., Ref. [41]) and may mask associations with clinical characteristics or therapy effects, even though the exact mechanisms are not yet clear. The fact that analyses with rigorous exclusion criteria regarding those factors showed changed results for intervention-focused analyses, while no definite proof, corroborates the necessity to control for these factors.
  - Non-systematic, open-ended assessment of medication intake (which, additionally, only was done in ∼60% of the participants) led to medication/medication changes during the intervention not being robust information for in-depth analyses. Especially an underreporting of “routine medication” such as topical corticosteroid and hormonal contraceptive use is probable, the former of which is a typical exclusion criterion for HCC studies (e.g., Ref. [14,15,18]).
  - No assessment of pregnancy/lactation/breastfeeding/menopause, major rhythm changes (e.g., shift work), quality of sleep, or further health- and lifestyle-associated factors which may influence HCC (e.g., Ref. [41]).

b) Regarding methodological aspects
  - Temporal mismatches between HCC and clinical questionnaires. Each of the sampled 3 cm hair strands roughly Table 3 reflects a 3-month period of cortisol secretion [47], whereas the HRSD [43] and the ERSQ [45] cover a one-week and the BDI-II a two-week interval [44]. Importantly, this is a typical issue also in the available literature. Further, available studies differ with regard to hair length assessed (3 cm, e.g., in Ref. [18,28] vs. 1 cm in, e.g., Ref. [17,29]). Moreover, the respective 3-month secretion periods analyzed here correspond with the three months before baseline (T1), the whole intervention phase (T6) and months 2-4 of subsequent individual psychotherapy (T11). Longer follow-up periods after the ART, and also after subsequent individual psychotherapy, would have been preferable. This is a common problem in psychotherapy research, and also prevalent in the only other study on HCC changes during a psychotherapeutic intervention in depressive disorders [31].

**Table 3:** Logistic regression analyses on the predictive value of baseline HCC on remission status at T6 and T11, with baseline clinical characteristics as covariate, for the sample following rigorous exclusion criteria.

Dependent	Predictor	B	SE	Wald Χ^2^	*p*	*OR*
T1 – T6: HRSD Remission status – ART & CFC (*n* = 47)	Baseline HRSD Score	0.18	0.07	6.50	0.011	1.19
	Baseline HCC	0.03	0.38	0.63	0.426	1.03

T1 – T6: HRSD Remission status – ART (*n* = 21)	Baseline HRSD Score	0.18	0.10	3.28	0.070	1.20
	Baseline HCC	0.09	0.09	1.09	0.296	1.09

T1 – T11: HRSD Remission status – ART & CFC (*n* = 40)	Baseline HRSD Score	0.04	0.05	0.69	0.406	1.04
	Baseline HCC	0.02	0.03	0.48	0.490	1.02

T1 – T11: HRSD Remission status – ART (*n* = 17)	Baseline HRSD Score	0.07	0.07	0.82	0.366	1.07
	Baseline HCC	−0.02	0.04	0.15	0.697	0.98

T1 – T6: BDI-II Remission status – ART & CFC (*n* = 40)	Baseline BDI-II Score	0.08	0.03	0.05	816	1.01
	Baseline HCC	0.12	0.09	1.91	0.167	1.13

T1 – T6: BDI-II Remission status – ART (*n* = 18)	Baseline BDI-II Score	−0.06	0.05	1.04	0.307	0.95
	Baseline HCC	0.04	0.09	0.23	0.628	1.04

T1 – T11: BDI-II Remission status – ART & CFC (*n* = 31)	Baseline BDI-II Score	0.05	0.04	1.37	0.242	1.05
	Baseline HCC	0.02	0.04	0.27	0.604	1.02

T1 – T11: BDI-II Remission status – ART (*n* = 24)	Baseline BDI-II Score^a^	n.a.	n.a.	n.a.	n.a.	n.a.
	Baseline HCC^a^	n.a.	n.a.	n.a.	n.a.	n.a.

ART = Affect Regulation Training condition, CFC = common-factor-control condition, HRSD = Hamilton Rating Scale for Depression, BDI-II = Beck Depression Inventory II; T1 = before the ten-week group intervention, T6 = ∼four weeks post-intervention (T1 + ∼12 weeks), T11 = ∼four months after the start of individual psychotherapy (T1 + ∼16-19 weeks)); ^a^ n.a = not available due to only *n* = 1 not having remitted.
  - Long storage time of hair samples. Although evidence on the impact on HCC is mixed (e.g., Ref. [58]; vs. [59]), and storage time neither differed between conditions, nor was associated with HCC in the present sample, current recommendations for larger/longitudinal studies advise to analyze hair samples at least once per year – albeit at the cost of introducing batch-related variability.
  - Hair analyses conducted in 2014. The protocol slightly differed from today's gold standard, most notably as CLIA instead of liquid chromatography tandem mass spectrometry (LC-MS/MS) was used. However, as assured by the analyzing lab (personal communication, November 20, 2023), the HCC data can be considered valid, and corresponds well with data from another study on individuals with major depressive disorder analyzed in the same timeframe and with the same CLIA methodology [14].
  - Exploratory nature of analyses and reduced statistical power following rigorous exclusion criteria. Although the original sample was relatively well-powered, the strict procedure led to considerable loss of power, limiting the validity of the results.

While the broad inclusion criteria are a crucial strength of the study regarding the primary study aim, proving the effectiveness of the ART under routine clinical conditions [40], they encumber the present, secondary HCC analyses. However, it must be noted that apart from analysis batch and BMI, none of the possible confounders assessed were associated with HCC at baseline. Potentially, this can, again, be explained by complex and non-linear interaction patterns of clinical and confounding effects that are impossible to discern in the current sample.

However, there is also a second possible interpretation of the findings: HCC, with the current expertise available, may, contrary to long-held hopes and expectations, not (yet) be suitable as a robust biomarker for psychopathology and psychotherapy effects under routine clinical conditions – a fate it may share with many other candidate biomarkers studied in the field (for a review, see, e.g., Ref. [9]). The few and inconsistent findings on cross-sectional and predictive associations in this study (that mostly would not survive corrections for multiple testing) align with the majority of previous studies also not reporting robust associations with clinical symptomatology [14,18,20,26,29,30] or therapy outcomes ([31]; but [32]) in depressive disorders. Moreover, in the literature, a considerable publication bias is likely, with null results (pertaining to whole datasets or singular measures) less frequently published than clear positive or negative findings. We do not wish to exempt the current study, given that it took more than ten years to report the less-than definite results. Thus, it is time to discuss potential reasons – beyond confounding factors – why HCC may not – as of yet - be suitable as a diagnostic, prognostic, or intervention-related biomarker.

With regard to diagnostics, most notably, HCC are generally considered a biomarker of stress-related HPA axis activity (e.g., Ref. [3,4]), associated more closely with arousal than with quality of affect, and thus, possibly, rather with transdiagnostic than disorder-specific processes. Specifically, depressive disorders show both profiles of increased (e.g., psychomotor agitation, sleep disturbances) and decreased arousal (e.g., psychomotor retardation, loss of interest/motivation, fatigue; [8]) – in addition to many other clinical facets. Previous studies have, for example, identified HCC differences in atypical vs. non-atypical depression [22] or first-onset vs. recurrent episode [28,29]. Additionally, length of episodes, depression-affected lifetime, individual fluctuation pattern, previous experiences with psychotherapy, or perceived disruption by the episode may play a role. Thus, HCC may rather bear potential as an additional source of information, potentially over the course of interventions, and not a precise diagnostic criterion. However, along the same lines, intervention success does not necessarily mean immediate/linear stress reduction. On the contrary, symptom improvement may take time even after the end of an intervention, and may cause cognitive, behavioral, and interpersonal changes and a need to adapt to those changes. All this may decrease, but also increase subjective stress, and may lead to vastly different profiles of HPA axis activity during and after treatment. Furthermore, it, so far, remains unclear whether HPA axis alterations are reversible consequences of psychopathology or rather pre-existing vulnerability factors, remaining unchanged even after successful intervention.

While this may seem disheartening, it is exactly why HCC and other candidate biomarkers may offer valuable potential in clinical research - for disentangling different profiles of symptomatology and therapy effects and thereby deepening the understanding of psychopathological processes. However, it remains a possibility that HPA axis changes in clinical conditions are so nuanced and complex that effects only emerge in large samples, which could mean they may never serve as a biomarker at the individual level. Looking ahead, while the last 15 years of research have yielded important insights, the initial goal of establishing a biomarker ready for clinical implementation seems far in the future [9]. However, these insights open research avenues that promise progress toward this goal:

First, for HCC research, and biomarker studies in general, clearer guidelines endorsed by the field are needed. While the present study was conducted from 2010 to 2014, and the past 15 years have seen great progress in identifying factors influencing HCC (e.g., Ref. [3,13]), procedures regarding confounding variables, analysis steps, and statistical preprocessing still differ considerably between laboratories, are passed down verbally, and have barely been systematically evaluated. Thus, also the (rigorous and minimum necessary) exclusion criteria in this study's preprocessing of the HCC data are open to debate. They were chosen based on the available literature on confounding and frequently controlled-for influences in HCC research (for an overview, see, e.g., Ref. [3,4,13,41]), the expertise of the associated researchers and laboratory, and the rationale of contrasting a rigorous and a more moderate approach with regard to the robustness of the findings. The past years have seen increased availability of guidelines (e.g., on open science in psychoneuroendocrinology; [60]; HCC research in trauma/PTSD; [41]; or the Cortisol Awakening Response; [61]). However, recent systematic reviews have shown unsatisfactory and somewhat even decreasing study quality in psychoneuroendocrinological research [41,62]. This shows: HCC studies are, notwithstanding the relative ease of sampling, a complex undertaking requiring considerable expertise.

Second, high quality clinical research in highly selective, large populations targeting singular conditions (e.g., depressive disorders, obesity, hypertension) is needed. Longitudinal cohort studies diligently tracking individual clinical patterns of symptom severity, remission phases, stress, and lifestyle factors, would help to disentangle the multitude of interacting factors impacting biological processes in general and HPA axis activity in particular. This is no mean feat as many confounding influences are particularly prevalent in clinical conditions (e.g., medication/substance intake, psychiatric/physical comorbidities) and controlling for them, naturally, comes at the cost of ecological validity. Here, an initial step might be to focus on conditions with a closer link to HPA axis activity (e.g., endocrine disorders; [63]) or clearer index events (e.g., psychotraumatological or postpartum research).

Finally, we urge translational researchers not to be discouraged by null results. In line with open science principles, null results are as valuable as spectacular findings, in that they teach crucial things – that a method requires refinement, a sample was underpowered, but also, and most importantly – that a theoretical assumption was not, or not robustly, visible in the analyzed sample. Thus, we want to encourage researchers to clean out their desks, sharing also “unspectacular” or “inconclusive” findings to advance the field [60].

In conclusion, although the present study, to the best of our knowledge, provides the first evidence for HCC decrease over a psychotherapeutic intervention in depressive disorders, this emerged only under rigorous control of potential confounders. This demonstrates the difficulties association with clinical-psychoneuroendocrinological research, but also highlights the progress made over the past 15 years. While the early enthusiasm that HCC analyses would soon be implemented in routine clinical practice has not yet been fulfilled, the next steps of research are to disentangle the complex mechanisms of HPA axis activity in psychiatric conditions. Much of what we know today stems from the pioneering work of Dirk Hellhammer, Clemens Kirschbaum, and the many researchers who have followed in their footsteps. However, this does not mean to blindly apply HCC in clinical settings, but rather to diligently and methodologically soundly advance research towards the goal of psychoneuroendocrinologically informed psychotherapy – one step at a time.

## CRediT authorship contribution statement

**Lena Schindler-Gmelch:** Writing – original draft, Visualization, Investigation, Formal analysis, Data curation, Conceptualization, Methodology. **Lukas Moses Fuhrmann:** Writing – review & editing, Investigation, Formal analysis, Data curation, Methodology. **Susann Steudte-Schmiedgen:** Writing – review & editing, Project administration, Methodology. **Matthias Berking:** Writing – review & editing, Supervision, Resources, Project administration, Methodology, Investigation, Funding acquisition, Conceptualization.

## Data statement

Study data and analysis code are openly available in OSF at https://osf.io/4pkhv/overview?view_only=b7f8b79226a24f039d8522714a4aa479.

## Declaration of generative AI and AI-assisted technologies in the manuscript preparation process

During the preparation of this work the author(s) used the generative AI tool ChatGPT for language editing and supporting in the development and troubleshooting of the code for statistical analyses. After using this tool/service, the author(s) reviewed and edited the content as needed and take(s) full responsibility for the content of the published article.

## Funding

This research was funded by the German Research Foundation (DFG, grant numbers BE4510/3-2; HI456/6-2, KI537/33-2 to Matthias Berking).

## Declaration of competing interest

The authors declare that they have no known competing financial interests or personal relationships that could have appeared to influence the work reported in this paper.
