# Supplementary material for: Hair cortisol in a naturalistic sample of outpatients with depressive disorders – A secondary analysis of a randomized-controlled trial and a status update on psychoneuroendocrine markers for clinical practice
Source: Compr Psychoneuroendocrinol. 2026 Sep 24;28:100380. doi: 10.1016/j.cpnec.2026.100380 (PMC13660528; doi:10.1016/j.cpnec.2026.100380)
Supplement: Supplementary file 1 [file mmc1.docx]

**Supplementary Material**

Supplement A: Demographic, clinical, and hair-related characteristics of the three study conditions following minimum necessary exclusion criteria for HCC analyses (*n* = 126).

|  | **Intervention condition** | | |  |  |  |
| --- | --- | --- | --- | --- | --- | --- |
|  | **ART**  *n* = 42 | **CFC**  *n* = 42 | **WLC**  *n* = 42 | Test statistic | *p*-value | Effect size |
| **Age (*M*, *SD*)** | 39.36 (13.95) | 35.64 (12.39) | 40.24 (12.97) | *F*(2, 123) = 1.45 | .238 | η^2^_p_ = .023 |
| **Female gender (*n*, *%*)** | 31 (73.8) | 29 (69.0) | 31 (73.8) | χ²(2) = 0.32 | .854 | *V* = .050 |
| **Migration background**  **(*n*, *%*)**^a^ | 3 (7.1) | 3 (7.1) | 2 (5.0) | χ²(2) = 0.21 | .902 | *V* = .041 |
| **Marital status** |  |  |  | χ²(8) = 7.94 | .440 | *V* = .177 |
| Single (*n*, %) | 9 (21.4) | 12 (28.6) | 9 (21.4) |  |  |  |
| In a relationship (*n*, %) | 15 (35.7) | 16 (38.1) | 11 (26.2) |  |  |  |
| Married, living together (*n*, %) | 11 (26.2) | 10 (23.8) | 17 (40.5) |  |  |  |
| Married, living separately (*n*, %) | 2 (4.8) | 0 (0) | 3 (7.1) |  |  |  |
| Divorced (*n*, %) | 5 (11.9) | 4 (9.%) | 2 (4.8) |  |  |  |
| **Body Mass Index**  **(BMI; *M*, *SD*)**^b^ | 27.44 (7.97) | 27.33 (5.12) | 26.68 (6.84) | *F*(2, 96) = 0.12 | .885 | η^2^_p_ = .003 |
| **BMI Categories**^b^ |  |  |  | χ²(10) = 14.67 | .145 | *V* = .272 |
| Underweight  (<18.5; *n*, %) | 2 (6.1) | 0 (0) | 1 (2.9) |  |  |  |
| Normal BMI  (18.5 – 24.9; *n*, %) | 15 (45.5) | 12 (37.5) | 19 (55.9) |  |  |  |
| Overweight  (25 – 29.9; *n*, %) | 6 (18.2) | 13 (40.6) | 4 (11.8) |  |  |  |
| Obesity Class I  (30 – 34.9; *n*, %) | 4 (12.1) | 5 (15.6) | 4 (11.8) |  |  |  |
| Obesity Class II  (35 – 39.9; *n*, %) | 2 (6.1) | 2 (6.3) | 4 (11.8) |  |  |  |
| Obesity Class III  (≥ 40; *n*, %) | 4 (12.1) | 0 (0) | 2 (5.9) |  |  |  |
| **Smoking (*n*, *%*)**^c^ | 10 (30.3) | 8 (24.2) | 11 (32.4) | χ²(2) = 0.58 | .750 | *V* = .076 |
| > 10 cigarettes/day  (*n*, *%*)^d^ | 3 (7.1) | 4 (9.5) | 4 (9.5) | χ²(2) = 0.20 | .905 | *V* = .040 |
| **Alcoholic drinks/week**  **(*M*, *SD*)**^e^ | 3.45 (5.31) | 3.77 (4.23) | 4.22 (4.82) | *F*(2, 98) = 0.22 | .805 | η^2^_p_ = .004 |
| “heavy drinkers”  (*n*, *%*)^e, f^ | 2 (5.9) | 4 (11.8) | 4 (12.1) | χ²(2) = 0.93 | .628 | *V* = .096 |
| **Hair washing frequency: Washes per week (*M*, *SD*)**^g^ | 4.43 (1.86) | 5.04 (1.84) | 4.73 (1.74) | *F*(2, 117) = 3.71 | .329 | η^2^_p_ = .019 |
| **Physical comorbidities**  **(*n*, %)**^c^ | 17 (51.5) | 13 (39.4) | 13 (38.2) | χ²(2) = 0.48 | .121 | *V* = .115 |
| Hypertension (*n*, %)^h^ | 4 (12.1) | 8 (25.0) | 3 (9.1) |  |  |  |
| Disease of the respiratory system (e.g., asthma, allergy; *n*, %)^h^ | 4 (12.1) | 2 (6.3) | 2 (6.1) |  |  |  |
| Diabetes (*n*, %)^h^ | 6 (18.2) | 2 (6.3) | 1 (3.0) |  |  |  |
| Thyroid Disease (*n*, %)^h^ | 2 (6.1) | 2 (6.3) | 3 (9.1) |  |  |  |
| Other (*n*, %)^h, i^ | 7 (21.2) | 1 (3.1) | 6 (18.2) |  |  |  |
| **Medication (*n*, %)**^j^ | 12 (41.4) | 14 (58.3) | 9 (39.1) | χ²(2) = 2.16 | .340 | *V* =.168 |
| Psychiatric medication (*n*, %)^j^ | 8 (27.6) | 11 (45.8) | 9 (39.1) | χ²(2) = 1.95 | .377 | *V* = .160 |
| **Primary diagnosis according to SCID (*n*, %)** |  |  |  | χ²(12) = 6.30 | .906 | *V* = .157 |
| F32.0: Mild depressive episode (*n*, %) | 4 (9.5) | 2 (4.8) | 2 (4.8) |  |  |  |
| F32.1: Moderate depressive episode (*n*, %) | 6 (14.3) | 9 (21.4) | 7 (16.7) |  |  |  |
| F33.0: Recurrent depressive disorder, current episode mild (*n*, %) | 11 (26.2) | 9 (23.8) | 13 (31.0) |  |  |  |
| F33.1: Recurrent depressive disorder, current episode moderate (*n*, %) | 18 (42.9) | 16 (38.1) | 18 (42.9) |  |  |  |
| F33.2: Recurrent depressive disorder, current episode severe without psychotic symptoms (*n*, %) | 2 (4.8) | 2 (4.8) | 0 (0) |  |  |  |
| F33.9: Recurrent depressive disorder, unspecified (*n*, %) | 0 (0) | 2 (4.8) | 1 (2.4) |  |  |  |
| F34.1: Dysthymia (*n*, %) | 1 (2.4) | 1 (2.4) | 1 (2.4) |  |  |  |
| **Current comorbid diagnoses according**  **to SCID (*n*, %)** | 24 (57.1) | 24 (57.1) | 25 (59.5) | χ²(2) = 0.65 | .968 | *V* = .023 |
| F3: Mood [affective] disorders (*n*, %) | 5 (11.9) | 4 (9.5) | 5 (11.9) |  |  |  |
| F4: Neurotic, stress-related and somatoform disorders (*n*, %) | 19 (45.2) | 18 (42.9) | 19 (45.2) |  |  |  |
| F5: Behavioural syndromes associated with physiological disturbances and physical factors (*n*, %) | 3 (7.1) | 6 (14.3) | 6 (14.3) |  |  |  |
| Other (*n*, %)^k^ | 1 (2.4) | 1 (2.4) | 1 (2.4) |  |  |  |
| **Number of current comorbid diagnoses according to SCID (*M, SD)*** | 0.74 (0.73) | 0.79 (0.87) | 0.81 (0.86) | *F*(2, 123) = 0.08 | .922 | η^2^_p_ = .001 |
| **Number of previous depressive episodes**  **(*M*, *SD)***^l^ | 1.82 (1.64) | 1.87 (1.79) | 2.09 (1.53) | *F*(2, 101) = 0.25 | .780 | η^2^_p_ = .005 |
| **Age at first episode (*M*, *SD)^m^*** | 26.43 (10.15) | 25.39 (11.79) | 28.77 (12.47) | *F*(2, 105) = 0.81 | .446 | η^2^_p_ = .015 |
| **T1 HRSD Score (*M, SD)^n^*** | 20.97 (7.81) | 19.95 (6.77) | 19.26 (7.93) | *F*(2, 112) = 0.51 | .605 | η^2^_p_ = .009 |
| **T1 BDI-II Score (*M, SD)^o^*** | 29.39 (11.10) | 27.18 (11.10) | 23.80 (10.63) | *F*(2, 99) = 2.14 | .123 | η^2^_p_ = .041 |
| **T1 ERSQ Score (*M, SD)^p^*** | 46.28 (20.21) | 47.45 (17.62) | 46.51 (17.76) | *F*(2, 94) = 0.04 | .961 | η^2^_p_ = .001 |
| **Season of T1 hair sampling (*n*, %)** |  |  |  | χ²(6) = 2.20 | .900 | *V* = .132 |
| Spring (*n*, %) | 13 (31.0) | 14 (33.3) | 16 (38.1) |  |  |  |
| Summer (*n*, %) | 10 (23.8) | 8 (19.0) | 11 (26.2) |  |  |  |
| Autumn (*n*, %) | 15 (35.7) | 15 (35.7) | 10 (23.8) |  |  |  |
| Winter (*n*, %) | 4 (9.5) | 5 (11.9) | 5 (11.9) |  |  |  |
| **Storage time of T1 hair samples in days *(M, SD)*** | 712.98 (219.54) | 708.36 (224.99) | 726.43 (220.81) | *F*(2, 123) = 0.08 | .928 | η^2^_p_ =.001 |
| **Storage time of T6 hair samples in days *(M, SD)***^q^ | 616.86 (223.71) | 607.37 (221.28) | 588.15 (205.91) | *F*(2, 94) = 0.14 | .867 | η^2^_p_ =.003 |
| **Storage time of T11 hair samples in days *(M, SD)****^r^* | 511.60 (220.50) | 503.57 (212.52) | 472.77 (195.41) | *F*(2, 66) = 0.23 | .795 | η^2^_p_ = .007 |
| **Samples analyzed in first batch (*n*, %)** | 16 (38.1) | 15 (35.7) | 16 (38.1) | χ²(2) = 0.07 | .967 | *V* = .023 |

ART = Affect Regulation Training condition, CFC = common-factor-control condition, WLC = waitlist control condition, BMI = body mass index, SCID = Structured Clinical Interview for DSM-IV Axis I and II Disorders, HRSD = Hamilton Rating Scale for Depression, range = 0 - 75, BDI-II = Beck Depression Inventory II, range = 0 - 63, ERSQ = Emotion Regulation Skills Questionnaire, range = 0 - 108; T1 = before the ten-week group intervention, T6 = ~four weeks post-intervention (T1 + ~12 weeks), T11 = ~four months after the start of individual psychotherapy (T1 + ~16-19 weeks); ^a^ *n* = 42, *n* = 42, *n* = 40, respectively; ^b^ *n* = 33, *n* = 32, *n* = 34, respectively; ^c^ *n* = 33, *n* = 33, *n* = 34, respectively; ^d^ *n* = 32, *n* = 32, *n* = 33, respectively; ^e^ *n* = 34, *n* = 34, *n* = 33, respectively; ^f^ “heavy drinkers” = >14 alcoholic drinks/week for men, >7 alcoholic drinks/week for women according to the National Institute on Alcohol Abuse and Alcoholism [NIAAA]; ^g^ *n* = 41, *n* = 41, *n* = 38, respectively; ^h^ *n* = 33, *n* = 32, *n* = 33, respectively; ^i^ Other = musculoskeletal/rheumatological (*n* = 4), gastrointestinal (*n* = 2), neurological (*n* = 1), dermatological/immunological (*n* = 1), gynecological/endocrinological (*n* = 1), vascular/hematological (*n* = 1), craniofacial/temporomandibular (*n* = 1) conditions; ^j^ *n* = 29, *n* = 24, *n* = 23, respectively; ^k^ Other = F1: Mental and behavioural disorders due to psychoactive substance use (*n* = 1), F6: Disorders of adult personality and behaviour (*n* = 1), F9: Behavioural and emotional disorders with onset usually occurring in childhood and adolescence (*n* = 1); ^l^ *n* = 34, *n* = 38, *n* = 32, respectively; ^m^ *n* = 35, *n* = 38, *n* = 35, respectively; ^n^ = 39, *n* = 38, *n* = 38, respectively; ^o^ *n* = 36, *n* = 36, *n* = 30, respectively; ^p^ *n* = 34, *n* = 34, *n* = 29, respectively; ^q^ *n* = 29, *n* = 35, *n* = 33, respectively; ^r^ *n* = 20, *n* = 23, *n* = 26, respectively

Supplement B. Robust linear regression analyses on the predictive value of baseline HCC on clinical characteristics at T6 and T11, with baseline clinical characteristics as covariate for the sample following minimum necessary exclusion criteria

| Dependent | Predictor | B [KI] | SE | Beta | *p* |
| --- | --- | --- | --- | --- | --- |
| T1 – T6: HRSD Change Score – ART & CFC (*n* = 66) | Baseline HRSD Score | -0.36  [-0.62; -0.09] | 0.13 | -.33 | .008 |
|  | Baseline HCC | 0.05  [-0.05; 0.15] | 0.05 | .13 | .310 |
|  |  |  |  |  |  |
| T1 – T6: HRSD Change Score – ART (*n* = 32) | Baseline HRSD Score | -0.23  [-0.53; 0.06] | 0.15 | -.25 | .123 |
|  | Baseline HCC | 0.03  [-0.11; 0.18] | 0.07 | .07 | .653 |
|  |  |  |  |  |  |
| T1 – T11: HRSD Change Score – ART & CFC (*n* = 55) | Baseline HRSD Score | -0.57  [-0.89; -0.26] | 0.16 | -.46 | < .001 |
|  | Baseline HCC | 0.06  [-0.05; 0.18] | 0.06 | .13 | .298 |
|  |  |  |  |  |  |
| T1 – T11: HRSD Change Score – ART (*n* = 25) | Baseline HRSD Score | -0.54  [-0.97; -0.11] | 0.22 | -.50 | .013 |
|  | Baseline HCC | -0.06  [-0.29; 0.18] | 0.12 | -.01 | .625 |
|  |  |  |  |  |  |
| T1 – T6: BDI-II Change Score – ART & CFC (*n* = 53) | Baseline BDI-II Score | -0.34  [-0.55; -0.13] | 0.11 | -.34 | .001 |
|  | Baseline HCC | 0.01  [-0.12; 0.13] | 0.06 | .01 | .926 |
|  |  |  |  |  |  |
| T1 – T6: BDI-II Change Score – ART (*n* = 26) | Baseline BDI-II Score | -0.37  [-0.71; -0.02] | 0.18 | -.35 | .036 |
|  | Baseline HCC | 0.04  [-0.28; 0.37] | 0.17 | .04 | .801 |
|  |  |  |  |  |  |
| T1 – T11: BDI-II Change Score – ART & CFC (*n* = 47) | Baseline BDI-II Score | -0.49  [-0.82; -0.17] | 0.17 | -.44 | .003 |
|  | Baseline HCC | 0.05  [-0.14; 0.24] | 0.1 | .07 | .632 |
|  |  |  |  |  |  |
| T1 – T11: BDI-II Change Score – ART (*n* = 24) | Baseline BDI-II Score | -0.32  [-0.74; 0.11] | 0.22 | -.30 | .144 |
|  | Baseline HCC | 0.04  [-0.30; 0.38] | 0.18 | .05 | .823 |
|  |  |  |  |  |  |
| T1 – T6: ERSQ Change Score –  ART & CFC (*n* = 50) | Baseline ERSQ Score | 0.40  [0.15; 0.66] | 0.13 | .42 | .002 |
|  | Baseline HCC | 0.08  [-0.18; 0.34] | 0.13 | .08 | .526 |
|  |  |  |  |  |  |
| T1 – T6: ERSQ Change Score –  ART (*n* = 24) | Baseline ERSQ Score | 0.27  [0; 0.55] | 0.14 | .37 | .051 |
|  | Baseline HCC | 0.14  [-0.31; 0.60] | 0.23 | .12 | .537 |
|  |  |  |  |  |  |
| T1 – T11: ERSQ Change Score – ART & CFC (*n* = 45) | Baseline ERSQ Score | 0.35  [0.08; 0.63] | 0.14 | .31 | .012 |
|  | Baseline HCC | 0.15  [-0.1; 0.40] | 0.13 | .14 | .246 |
|  |  |  |  |  |  |
| T1 – T11: ERSQ Change Score – ART (*n* = 22) | Baseline ERSQ Score | 0.28  [-0.02; 0.59] | 0.16 | .40 | .071 |
|  | Baseline HCC | -0.03  [-0.46; 0.39] | 0.22 | -.03 | .881 |

ART = Affect Regulation Training condition, CFC = common-factor-control condition, HRSD = Hamilton Rating Scale for Depression, BDI-II = Beck Depression Inventory II, ERSQ = Emotion Regulation Skills Questionnaire; T1 = before the ten-week group intervention, T6 = ~four weeks post-intervention (T1 + ~12 weeks), T11 = ~four months after the start of individual psychotherapy (T1 + ~16-19 weeks);

Supplement C. Logistic regression analyses on the predictive value of baseline HCC on remission status at T6 and T11, with baseline clinical characteristics as covariate for the sample following minimum necessary exclusion criteria for HCC analyses

| Dependent | Predictor | B | SE | Wald Χ^2^ | *p* | *OR* |
| --- | --- | --- | --- | --- | --- | --- |
| T1 – T6: HRSD Remission status – ART & CFC (*n* = 67) | Baseline HRSD Score | 0.17 | 0.06 | 7.86 | .005 | 1.18 |
|  | Baseline HCC | 0.04 | 0.04 | 1.37 | .241 | 1.04 |
|  |  |  |  |  |  |  |
| T1 – T6: HRSD Remission status – ART (*n* = 33) | Baseline HRSD Score | 0.13 | 0.07 | 3.61 | .058 | 1.14 |
|  | Baseline HCC | 0.07 | 0.06 | 1.46 | .227 | 1.07 |
|  |  |  |  |  |  |  |
| T1 – T11: HRSD Remission status – ART & CFC (*n* = 55) | Baseline HRSD Score | 0.08 | 0.04 | 2.91 | .088 | 1.08 |
|  | Baseline HCC | 0.02 | 0.02 | 0.72 | .395 | 1.02 |
|  |  |  |  |  |  |  |
| T1 – T11: HRSD Remission status – ART (*n* = 25) | Baseline HRSD Score | 0.07 | 0.06 | 1.38 | .241 | 1.07 |
|  | Baseline HCC | -0.01 | 0.03 | 0.08 | .781 | 0.99 |
|  |  |  |  |  |  |  |
| T1 – T6: BDI-II Remission status – ART & CFC (*n* = 53) | Baseline BDI-II Score | 0.19 | 0.33 | 0.32 | .570 | 1.02 |
|  | Baseline HCC | 0.17 | 0.09 | 3.47 | .063† | 1.18 |
|  |  |  |  |  |  |  |
| T1 – T6: BDI-II Remission status – ART (*n* = 26) | Baseline BDI-II Score | -0.01 | 0.04 | 0.04 | .833 | 0.99 |
|  | Baseline HCC | 0.15 | 0.14 | 1.12 | .289 | 1.16 |
|  |  |  |  |  |  |  |
| T1 – T11: BDI-II Remission status – ART & CFC (*n* = 47) | Baseline BDI-II Score | 0.07 | 0.03 | 3.86 | .050† | 1.07 |
|  | Baseline HCC | 0.05 | 0.04 | 1.49 | .223 | 1.05 |
|  |  |  |  |  |  |  |
| T1 – T11: BDI-II Remission status – ART (*n* = 24) | Baseline BDI-II Score | 0.23 | 0.18 | 1.64 | .200 | 1.26 |
|  | Baseline HCC | 0.77 | 0.47 | 2.71 | .100 | 2.16 |

ART = Affect Regulation Training condition, CFC = common-factor-control condition, HRSD = Hamilton Rating Scale for Depression, BDI-II = Beck Depression Inventory II; T1 = before the ten-week group intervention, T6 = ~four weeks post-intervention (T1 + ~12 weeks), T11 = ~four months after the start of individual psychotherapy (T1 + ~16-19 weeks);
